# Supplementary material for: Reduced serotonergic transmission alters sensitivity to cost and reward via 5-HT1A and 5-HT1B receptors in monkeys
Source: PLoS Biol. 2024 Jan 1;22(1):e3002445. doi: 10.1371/journal.pbio.3002445 (PMC10758260; doi:10.1371/journal.pbio.3002445)
Supplement: S1 Table — A tick indicates the monkey used the experiment. Species (J, Japanese; R, Resus); Sex (F, Female; M, Male); BW, body weight; CSF, cerebrospinal fluid; RS task, reward-size task; W/D task, work/delay task. (DOCX) [file pbio.3002445.s001.docx]

**Supporting Information**

**Table S1. Summary of subjects used in this study.**

| Monkey | Species | Sex | BW | Age (y) | 5-HT depletion | | 5-HTR blockade | | PET |
| --- | --- | --- | --- | --- | --- | --- | --- | --- | --- |
|  |  |  |  |  | CSF | RS task | RS task | W/D task |  |
| BI | R | M | 7.9 | 7 |  |  | ✔ |  |  |
| BO | R | M | 5.7 | 7 |  | ✔ | ✔ |  |  |
| CH | R | M | 7.3 | 4 |  |  |  |  | ✔ |
| DR | R | M | 5.7 | 5 |  |  |  |  | ✔ |
| KN | R | M | 6.1 | 12 |  | ✔ |  |  |  |
| KY | R | M | 5.9 | 11 |  |  |  | ✔ |  |
| MP | J | M | 7.7 | 7 |  |  |  | ✔ |  |
| PE | R | M | 4.5 | 6 | ✔ |  |  |  |  |
| RI | R | M | 5.5 | 5 |  |  |  |  | ✔ |
| SA | R | M | 6.1 | 5 | ✔ |  |  |  |  |
| ST | R | M | 6.2 | 12 |  | ✔ |  | ✔ |  |
| TK | R | M | 7.7 | 9 |  |  | ✔ |  |  |
| TO | R | M | 7.0 | 9 |  | ✔ | ✔ |  |  |
| TN | J | M | 7.5 | 5 |  |  |  |  | ✔ |
| TS | R | M | 5.1 | 8 |  |  |  |  | ✔ |
| Total | 15 |  |  |  | 2 | 4 | 4 | 3 | 5 |

A tick indicates the monkey used in the experiment. Species (J, Japanese; R, Resus); Sex (F, Female; M, Male); BW, body wight; CSF, cerebrospinal fluid; RS task, reward-size task; W/D task, work/delay task; PET, receptor mapping or occupancy measurement using positron emission tomography.
