# Supplementary material for: Reduced serotonergic transmission alters sensitivity to cost and reward via 5-HT1A and 5-HT1B receptors in monkeys
Source: PLoS Biol. 2024 Jan 1;22(1):e3002445. doi: 10.1371/journal.pbio.3002445 (PMC10758260; doi:10.1371/journal.pbio.3002445)
Supplement: S2 Table — Numbers indicate the number of treatments. (DOCX) [file pbio.3002445.s002.docx]

**S2 Table. Summary of drug treatment for each monkey**

|  | RS task | RS task | | | | W/D task | | | |
| --- | --- | --- | --- | --- | --- | --- | --- | --- | --- |
| Monkey | Depletion | 5-HT_1A_ | 5-HT_1B_ | 5-HT_2A_ | 5-HT_4_ | 5-HT_1A_ | 5-HT_1B_ | 5-HT_2A_ | 5-HT_4_ |
| BI |  |  | 4 | 4 | 4 |  |  |  |  |
| BO | 5 |  |  |  |  |  |  |  |  |
| KN | 1 | 4 | 4 | 4 |  |  |  |  |  |
| KY |  |  |  |  |  | 4 | 4 | 4 | 4 |
| MP |  |  |  |  |  | 4 | 4 | 4 | 4 |
| ST | 1 |  |  |  |  | 4 | 4 | 4 | 4 |
| TK |  | 4 | 4 |  | 4 |  |  |  |  |
| TO | 4 | 4 |  | 4 | 4 |  |  |  |  |

Numbers indicate the number of treatments.
