# Supplementary material for: Reduced serotonergic transmission alters sensitivity to cost and reward via 5-HT1A and 5-HT1B receptors in monkeys
Source: PLoS Biol. 2024 Jan 1;22(1):e3002445. doi: 10.1371/journal.pbio.3002445 (PMC10758260; doi:10.1371/journal.pbio.3002445)
Supplement: S3 Table — a(cond) and e(cond) indicate the random effects of conditions with 5-HT depletion on parameters a and e, respectively. The random effect of Model #3 was assumed to be normally distributed on a and e, independently. The random effect of Model #4 was assumed to be a 2D normal distribution on a and e: (1/acond, econd) ~ biNorm(0, ∑cond). BIC is a relative measure of quality for the models (#1–5). ΔBIC denotes the difference from the minimum BIC. (DOCX) [file pbio.3002445.s003.docx]

**S3 Table. Model comparison for the effect of 5-HT depletion on error rate (for Fig. 1E)**

| Model | | BIC | ΔBIC |
| --- | --- | --- | --- |
| #1 | *E = 1/a(cond) R* | 29.2 | 13.3 |
| #2 | *E = 1/a(cond) R + e* | 25.2 | 9.3 |
| #3 | *E = 1/a(cond) R + e(cond)* | 18.1 | 2.2 |
| **#4** | ***E = 1/a(cond) R + e(cond), with (1/a(cond), e(cond)) ~ biNorm*** | **15.9** | **0** |
| #5 | *E = 1/a R + e(cond)* | 22.2 | 6.3 |

*a*(*cond*) and *e*(*cond*) indicate the random effects of conditions with 5-HT depletion on parameters *a* and *e*, respectively. The random effect of Model #3 was assumed to normally distributed on a and e, independently. The random effect of Model #4 was assumed to be a two-dimensional normal distribution on *a* and *e*; (1/*a_cond_*, *e_cond_*) ~ biNorm(0, ∑_cond_). BIC is a relative measure of quality for the models (#1-5). ΔBIC denotes the difference from the minimum BIC.
