# Supplementary material for: Reduced serotonergic transmission alters sensitivity to cost and reward via 5-HT1A and 5-HT1B receptors in monkeys
Source: PLoS Biol. 2024 Jan 1;22(1):e3002445. doi: 10.1371/journal.pbio.3002445 (PMC10758260; doi:10.1371/journal.pbio.3002445)
Supplement: S4 Table — a(mk), a(cond), e(mk), and e(cond) indicate the random effects of 5-HT depletion conditions on parameters a and e, respectively. The probability distribution of the random effects were as follows: amk and acond were ~N(0,σ2mk) and ~N(0,σ2cond), respectively. (a’mk, e’mk) and (a’cond, e’cond) were ~biNorm(0, ∑mk) and ~biNorm(0, ∑cond), respectively. E, error rate; RT, reaction time; mk, monkey; cond, treatment condition (pCPA-day1, pCPA-day2, or control). BIC is a relative measure of quality for the models (#1–4). ΔBIC denotes the difference from the minimum BIC. (DOCX) [file pbio.3002445.s004.docx]

**S4 Table. Model comparison for the effect of 5-HT depletion on the relationship between error rate and RT in the reward-size task (for Fig 2D)**

| Model | | BIC | ΔBIC |
| --- | --- | --- | --- |
| #1 | *E = aRT + e* | 1768 | **18** |
| #2 | *E =* (*a* + *a'_mk_*)*RT + e + e'_mk_* | 1756 | **7** |
| #3 | *E =* (*a* + *a'_cond_*)*RT + e + e'_cond_* | 1764 | **15** |
| **#4** | ***E =* (*a* + *a'_mk_* + *a'_cond_*)*RT + e + e'_mk_ + e'_cond_*** | 1749 | **0** |

*a*(*mk*), *a*(*cond*), *e*(*mk*),and *e*(*cond*) indicate the random effects of 5-HT depletion conditions on parameters *a* and *e*, respectively. The probability distribution of the random effects were as follows; *a_mk_* and *a_cond_* were ~N(0,σ^2^_mk_) and ~N(0,σ^2^_cond_), respectively. (*a’_mk_, e’_mk_*) and (*a’_cond_, e’_cond_*) were ~biNorm(0, ∑_mk_) and ~biNorm(0, ∑_cond_), respectively. *E*, error rate; *RT*, reaction time; *mk*, monkey; *cond*, treatment condition (pCPA-day1, pCPA-day2 or control). BIC is a relative measure of quality for the models (#1-4). ΔBIC denotes the difference from the minimum BIC.
