# Supplementary material for: Reduced serotonergic transmission alters sensitivity to cost and reward via 5-HT1A and 5-HT1B receptors in monkeys
Source: PLoS Biol. 2024 Jan 1;22(1):e3002445. doi: 10.1371/journal.pbio.3002445 (PMC10758260; doi:10.1371/journal.pbio.3002445)
Supplement: S5 Table — a(cond) a(mk), a(cond_mk), e(cond), e(mk), and e(cond_mk) indicate the random effects of blocking 5-HTR on parameters a and e, respectively. The probability distribution of the random effects were as follows: acond, amk, and acond_mk were ~N(0,σ2cond), ~N(0,σ2mk), and ~N(0,σ2cond_mk), respectively. (a’cond, e’cond), (a’mk, e’mk), and (a’cond_mk, e’cond_mk) were ~biNorm(0, ∑cond), ~biNorm(0, ∑mk), and ~biNorm(0, ∑cond_mk), respectively. E, error rate; cond, treatment condition (antagonist or control); mk, monkey; cond_mk, interaction of treatment condition and monkey. BIC is a relative measure of quality for the models (#1–30). ΔBIC denotes the difference from the minimum BIC. (DOCX) [file pbio.3002445.s005.docx]

**S5 Table. Model comparison for the effect of 5-HTR blockade on error rate in the reward-size task (for Fig. 4)**

|  |  | 5-HT_1A_ | | 5-HT_1B_ | | 5-HT_2A_ | | 5-HT_4_ | |
| --- | --- | --- | --- | --- | --- | --- | --- | --- | --- |
| Model | | BIC | ΔBIC | BIC | ΔBIC | BIC | ΔBIC | BIC | ΔBIC |
| #1 | *E =* 1*/aR* | 216 | 66 | 152 | 51 | 134 | 34 | 97 | 34 |
| #2 | *E =* 1*/aR + e* | 217 | 67 | 144 | 43 | 124 | 24 | 75 | 13 |
| #3 | *E =* 1*/aR +* 0 | 216 | 66 | 141 | 40 | 124 | 24 | 76 | 13 |
| #4 | *E =* 1*/aR + e*(*cond)* | 195 | 44 | 142 | 41 | 124 | 24 | 76 | 14 |
| #5 | *E = 1/aR + e(cond), with (1/a(mk), e(mk)) ~ biNorm* | 196 | 46 | 118 | 17 | 105 | 4 | 79 | 17 |
| #6 | *E = 1/a(mk)R + e(cond, mk)* | 194 | 44 | 116 | 16 | 104 | 3 | 76 | 14 |
| #7 | *E = 1/aR + e(cond, mk)* | 191 | 41 | 129 | 28 | 106 | 5 | 78 | 15 |
| #8 | *E = 1/a(mk)R + e(cond)* | 192 | 41 | 113 | 13 | 105 | 5 | 73 | 11 |
| #9 | *E =* 1*/a*(*cond*)*R + e* | 197 | 46 | 142 | 41 | 124 | 24 | 72 | 10 |
| #10 | *E = 1/a(cond)R + e , with (1/a(mk), e(mk)) ~ biNorm* | 200 | 50 | 109 | 9 | 105 | 4 | 72 | 10 |
| #11 | *E = 1/a(cond, mk)R + e(mk)* | 198 | 47 | 109 | 8 | 104 | 3 | 69 | 7 |
| #12 | *E = 1/a(cond)R + e(mk)* | 195 | 44 | 127 | 26 | 106 | 5 | 73 | 10 |
| #13 | *E = 1/a(cond, mk)R + e* | 195 | 45 | 106 | 5 | 105 | 5 | 66 | 3 |
| #14 | *E =* 1*/a*(*cond*)*R + e*(*cond*)*, with* (1*/a*(*cond*)*, e*(*cond*)) *~ biNorm* | 199 | 49 | 148 | 47 | 131 | 30 | 79 | 16 |
| #15 | *E = 1/a(cond)R + e(cond), with (1/a\|(cond, mk), e\|(cond, mk)) ~ biNorm* | 199 | 49 | 113 | 13 | 111 | 11 | 79 | 16 |
| #16 | *E = 1/a(cond, mk)R + e(cond, mk), with (1/a(cond), e\|(cond)) ~ biNorm* | 197 | 47 | 113 | 12 | 110 | 10 | 75 | 13 |
| #17 | *E = 1/a(cond)R + e(cond, mk), with (1/a(cond), e\|(cond)) ~ biNorm* | 194 | 44 | 133 | 32 | 112 | 12 | 79 | 17 |
| #18 | *E = 1/a(cond, mk)R + e(cond), with (1/a(cond), e(cond)) ~ biNorm* | 195 | 45 | 111 | 10 | 112 | 11 | 72 | 10 |
| #19 | *E =* 1*/a*(*cond*)*R + e*(*cond*) | 197 | 47 | 145 | 44 | 127 | 27 | 75 | 13 |
| #20 | *E = 1/a(cond)R + e(cond), with (1/a(mk), e(mk)) ~ biNorm* | 198 | 47 | 112 | 12 | 108 | 8 | 75 | 12 |
| #21 | *E = 1/a(cond, mk)R + e(cond, mk)* | 196 | 45 | 112 | 11 | 107 | 6 | 72 | 10 |
| #22 | *E = 1/a(cond)R + e(cond, mk)* | 193 | 42 | 130 | 29 | 109 | 9 | 76 | 14 |
| #23 | *E = 1/a(cond, mk)R + e(cond)* | 194 | 43 | 109 | 8 | 108 | 8 | 69 | 7 |
| #24 | *E = 1/a(mk)R + e(mk), with (1/a, e) ~ biNorm* | 214 | 64 | 116 | 16 | 102 | 1 | 82 | 19 |
| #25 | *E = 1/a(mk)R + e(mk)* | 211 | 61 | 115 | 14 | **100** | **0** | 79 | 16 |
| #26 | *E = 1/aR + e(mk)* | 208 | 58 | 126 | 25 | 103 | 2 | 78 | 16 |
| #27 | *E = 1/a(mk)R + e* | 208 | 58 | 112 | 11 | 102 | 2 | 75 | 13 |
| #28 | *E = 1/aR + e(cond_mk)* | 177 | 26 | 131 | 30 | 110 | 10 | 76 | 14 |
| #29 | *E = 1/a(cond_mk)R + e(cond_mk) , with (1/a(cond_mk), e(cond_mk)) ~ biNorm* | **151** | **0** | 104 | 3 | 109 | 8 | 69 | 6 |
| #30 | *E = 1/a(cond_mk)R + e* | 186 | 36 | **101** | **0** | 108 | 8 | **62** | **0** |

*a(cond)* *a*(*mk*), *a*(*cond_mk*), *e(cond)*, *e*(*mk*), and *e*(*cond_mk*) indicate the random effects of blocking 5-HTR on parameters *a* and *e*, respectively. The probability distribution of the random effects were as follows; *a_cond_* , *a_mk_* and *a_cond_mk_* were ~N(0,σ^2^_cond_), ~N(0,σ^2^_mk_), and ~N(0,σ^2^_cond_mk_), respectively. (*a’_cond_, e’_cond_*), (*a’_mk_, e’_mk_*), and (*a’_cond_mk_, e’_cond_mk_*) were ~biNorm(0, ∑_cond_), ~biNorm(0, ∑_mk_), and ~biNorm(0, ∑_cond_mk_), respectively. *E*, error rate; *cond*, treatment condition (antagonist or control); *mk*, monkey; *cond_mk*, interaction of treatment condition and monkey. BIC is a relative measure of quality for the models (#1-30). ΔBIC denotes the difference from the minimum BIC.
