# Supplementary material for: Reduced serotonergic transmission alters sensitivity to cost and reward via 5-HT1A and 5-HT1B receptors in monkeys
Source: PLoS Biol. 2024 Jan 1;22(1):e3002445. doi: 10.1371/journal.pbio.3002445 (PMC10758260; doi:10.1371/journal.pbio.3002445)
Supplement: S6 Table — a(mk), a(cond), e(mk), and e(cond) indicate the random effects of 5-HT depletion on parameters a and e, respectively. The probability distribution of the random effects were as follows: acond and amk were ~N(0,σ2cond) and ~N(0,σ2mk), respectively. (a’cond, e’cond) and (a’mk, e’mk) were ~biNorm(0, ∑cond) and ~biNorm(0, ∑mk), respectively. E, error rate; RT, reaction time; mk, monkey; cond, treatment condition (antagonist or control). BIC is a relative measure of quality for the models. ΔBIC denotes the difference from the minimum BIC. (DOCX) [file pbio.3002445.s006.docx]

**S6 Table. Model comparison for the effect of 5-HTR blockade on the relationship between error rate and RT in the reward size task (for Fig. S4)**

|  |  | 5-HT_1A_ | | 5-HT_1B_ | | 5-HT_2A_ | | 5-HT_4_ | |
| --- | --- | --- | --- | --- | --- | --- | --- | --- | --- |
| Model | | BIC | ΔBIC | BIC | ΔBIC | BIC | ΔBIC | BIC | ΔBIC |
| #1 | *E = e* | 1893 | 231 | 1021 | 63 | 1367 | 128 | 880 | 34 |
| #2 | *E = aRT + e* | 1685 | 23 | 1016 | 58 | 1350 | 110 | 884 | 38 |
| #3 | *E =* (*a* + *a'_mk_*)*RT + e + e'_mk_* | 1696 | 33 | 966 | 8 | 1249 | 9 | 853 | 7 |
| #4 | *E =* (*a* + *a'_mk_*)*RT + e* | 1692 | 29 | **958** | **0** | **1240** | **0** | **846** | **0** |
| #5 | *E = aRT + e + e_mk_* | 1690 | 28 | 961 | 2 | 1243 | 3 | 848 | 2 |
| #6 | *E =* (*a* + *a'_cond_*)*RT + e + e'_cond_* | 1672 | 9 | 1038 | 79 | 1368 | 129 | 910 | 64 |
| #7 | *E =* (*a* + *a_cond_*)*RT + e* | **1662** | **0** | 1028 | 69 | 1363 | 123 | 899 | 54 |
| #8 | *E = aRT + e + e_cond_* | 1668 | 6 | 1027 | 69 | 1363 | 123 | 899 | 54 |
| #9 | *E =* (*a* + *a'_mk_* + *a'_cond_*)*RT + e + e'_mk_ + e'_cond_* | 1694 | 32 | 977 | 18 | 1277 | 37 | 865 | 19 |
| #10 | *E =* (*a* + *a'_mk_*)*RT + e + e'_mk_ + e'_cond_* | 1681 | 19 | 966 | 8 | 1271 | 31 | 856 | 10 |
| #11 | *E =* (*a* + *a'_mk_*)*RT + e + e_cond_* | 1679 | 16 | 969 | 10 | 1254 | 14 | 859 | 13 |
| #12 | *E =* (*a* + *a_mk_ + a'_cond_*)*RT + e + e'_cond_* | 1676 | 13 | 985 | 27 | 1271 | 31 | 875 | 29 |
| #13 | *E =* (*a* + *a_mk_ + a_cond_*)*RT + e* | 1668 | 5 | 975 | 16 | 1263 | 23 | 865 | 19 |
| #14 | *E =* (*a* + *a_mk_*)*RT + e + e_cond_* | 1673 | 11 | 962 | 4 | 1250 | 10 | 852 | 7 |
| #15 | *E =* (*a* + *a'_cond_*)*RT + e + e _mk_ + e'_cond_* | 1677 | 15 | 973 | 14 | 1256 | 16 | 860 | 15 |
| #16 | *E =* (*a* + *a_cond_*)*RT + e + e _mk_* | 1668 | 6 | 963 | 4 | 1249 | 9 | 852 | 6 |
| #17 | *E = aRT + e + e_mk_ + e_cond_* | 1673 | 11 | 963 | 4 | 1249 | 9 | 852 | 6 |

*a*(*mk*), *a*(*cond*), *e*(*mk*),and *e*(*cond*) indicate the random effects of 5-HT depletion on parameters *a* and *e*, respectively. The probability distribution of the random effects were as follows; *a_cond_* and *a_mk_* were ~N(0,σ^2^_cond_) and ~N(0,σ^2^_mk_), respectively. (*a’_cond_, e’_cond_*) and (*a’_mk_, e’_mk_*) were ~biNorm(0, ∑_cond_) and ~biNorm(0, ∑_mk_), respectively. *E*, error rate; *RT*, reaction time; *mk*, monkey; *cond*, treatment condition (antagonist or control). BIC is a relative measure of quality for the models. ΔBIC denotes the difference from the minimum BIC.
