# Supplementary material for: Reduced serotonergic transmission alters sensitivity to cost and reward via 5-HT1A and 5-HT1B receptors in monkeys
Source: PLoS Biol. 2024 Jan 1;22(1):e3002445. doi: 10.1371/journal.pbio.3002445 (PMC10758260; doi:10.1371/journal.pbio.3002445)
Supplement: S7 Table — CU and E0 indicate the remaining cost and the intercept, respectively. k(treat), k(type), e(treat), and e(type) denote the random effects of blocking 5-HT on parameters k and e, respectively. The probability distribution of the random effects were as follows: ktreat and ktype were ~N(0,σ2treat) and ~N(0,σ2type), respectively. (k’treat, e’treat) and (k’type, e’type) were ~biNorm(0, ∑treat) and ~biNorm(0, ∑type), respectively. E, error rate; treat, treatment condition (antagonist or control): type, trial type (delay or work). BIC is a relative measure of quality for the models (#1–25). ΔBIC denotes the difference from the minimum BIC. (DOCX) [file pbio.3002445.s007.docx]

**S7 Table. Model comparison for the effect of 5-HTR blockade on error rate in the work/delay task (for Fig. 5C)**

| 5-HT_1A_ blockades | | monkey KY | | monkey MP | | monkey ST | |
| --- | --- | --- | --- | --- | --- | --- | --- |
|  | Model | BIC | ΔBIC | BIC | ΔBIC | BIC | ΔBIC |
| #1 | *E = k CU + E*_0_ | 357 | 1 | 832 | 139 | 563 | 83 |
| #2 | *E = k CU + E*_0_ *+ E*_0_*\|treat* | 364 | 9 | 841 | 148 | 574 | 94 |
| #3 | *E = k CU + E*_0_ *+ E*_0_*\|type* | 369 | 13 | 729 | 36 | 512 | 32 |
| #4 | *E = k CU + E*_0_ *+ E*_0_*\|treat + E*_0_*\|type* | 369 | 13 | 734 | 41 | 540 | 60 |
| #5 | *E = k CU + E*_0_ *+ E*_0_*\|*(*treat * type*) | 360 | 4 | 833 | 140 | 566 | 85 |
| #6 | *E =* (*k + k\|treat*)*CU + E*_0_ | 362 | 7 | 729 | 36 | 502 | 22 |
| #7 | *E =* (*k + k\|treat*)*CU + E*_0_ *+ E*_0_*\|treat* | 371 | 15 | 716 | 23 | 510 | 30 |
| #8 | *E =* (*k + k\|treat*)*CU + E*_0_ *+ E*_0_*\|type* | 356 | 1 | 831 | 138 | 565 | 85 |
| #9 | *E =* (*k + k\|treat*)*CU + E*_0_ *+ E*_0_*\|treat + E*_0_*\|type* | 373 | 17 | 711 | 18 | 514 | 34 |
| #10 | *E =* (*k + k\|treat*)*CU + E*_0_ *+ E*_0_*\|*(*treat * type*) | 364 | 9 | 730 | 38 | 533 | 53 |
| #11 | *E =* (*k + k\|type*)*CU + E*_0_ | 360 | 5 | 725 | 32 | 531 | 51 |
| #12 | *E =* (*k + k\|type*)*CU + E*_0_ *+ E*_0_*\|treat* | **356** | **0** | 714 | 21 | **480** | **0** |
| #13 | *E =* (*k + k\|type*)*CU + E*_0_ *+ E*_0_*\|type* | 364 | 9 | 697 | 4 | 489 | 9 |
| #14 | *E =* (*k + k\|type*)*CU + E*_0_ *+ E*_0_*\|treat + E*_0_*\|type* | 378 | 22 | 713 | 20 | 520 | 40 |
| #15 | *E =* (*k + k\|type*)*CU + E*_0_ *+ E*_0_*\|*(*treat * type*) | 361 | 5 | 727 | 34 | 526 | 46 |
| #16 | *E =* (*k + k\|treat + k\|type*)*CU + E*_0_ | 360 | 5 | 720 | 27 | 503 | 23 |
| #17 | *E =* (*k + k\|treat + k\|type*)*CU + E*_0_ *+ E*_0_*\|treat* | 362 | 7 | 733 | 40 | 529 | 49 |
| #18 | *E =* (*k + k\|treat + k\|type*)*CU + E*_0_ *+ E*_0_*\|type* | 364 | 9 | 698 | 5 | 489 | 9 |
| #19 | *E =* (*k + k\|treat + k\|type*)*CU + E*_0_ *+ E*_0_*\|treat + E*_0_*\|type* | 360 | 4 | **693** | **0** | 484 | 4 |
| #20 | *E =* (*k + k\|treat + k\|type*)*CU + E*_0_ *+ E*_0_*\|*(*treat * type*) | 364 | 9 | 726 | 33 | 506 | 26 |
| #21 | *E =* (*k + k\|*(*treat * type*))*CU + E*_0_ | 360 | 5 | 728 | 35 | 530 | 50 |
| #22 | *E =* (*k + k\|*(*treat * type*))*CU + E*_0_ *+ E*_0_*\|treat* | 369 | 13 | 703 | 10 | 511 | 31 |
| #23 | *E =* (*k + k\|*(*treat * type*))*CU + E*_0_ *+ E*_0_*\|type* | 365 | 9 | 702 | 9 | 500 | 20 |
| #24 | *E =* (*k + k\|*(*treat * type*))*CU + E*_0_ *+ E*_0_*\|treat + E*_0_*\|type* | 360 | 4 | 719 | 26 | 485 | 4 |
| #25 | *E =* (*k + k\|*(*treat * type*))*CU + E*_0_ *+ E*_0_*\|*(*treat * type*) | 365 | 9 | 701 | 8 | 501 | 21 |

| 5-HT_1B_ blockades | | monkey KY | | monkey MP | | monkey ST | |
| --- | --- | --- | --- | --- | --- | --- | --- |
|  | Model | BIC | ΔBIC | BIC | ΔBIC | BIC | ΔBIC |
| #1 | *E = k CU + E*_0_ | 367 | 3 | 274 | 1 | 350 | 33 |
| #2 | *E = k CU + E*_0_ *+ E*_0_*\|treat* | 372 | 9 | 281 | 9 | 325 | 9 |
| #3 | *E = k CU + E*_0_ *+ E*_0_*\|type* | 377 | 13 | 284 | 12 | 329 | 13 |
| #4 | *E = k CU + E*_0_ *+ E*_0_*\|treat + E*_0_*\|type* | 377 | 13 | 285 | 13 | 329 | 13 |
| #5 | *E = k CU + E*_0_ *+ E*_0_*\|*(*treat * type*) | 367 | 4 | 276 | 4 | 338 | 21 |
| #6 | *E =* (*k + k\|treat*)*CU + E*_0_ | 371 | 7 | 277 | 5 | 355 | 38 |
| #7 | *E =* (*k + k\|treat*)*CU + E*_0_ *+ E*_0_*\|treat* | 380 | 16 | 285 | 13 | 363 | 47 |
| #8 | *E =* (*k + k\|treat*)*CU + E*_0_ *+ E*_0_*\|type* | **364** | **0** | 273 | 1 | **317** | **0** |
| #9 | *E =* (*k + k\|treat*)*CU + E*_0_ *+ E*_0_*\|treat + E*_0_*\|type* | 380 | 16 | 288 | 16 | 350 | 34 |
| #10 | *E =* (*k + k\|treat*)*CU + E*_0_ *+ E*_0_*\|*(*treat * type*) | 372 | 8 | 280 | 8 | 342 | 25 |
| #11 | *E =* (*k + k\|type*)*CU + E*_0_ | 368 | 4 | 277 | 5 | 321 | 4 |
| #12 | *E =* (*k + k\|type*)*CU + E*_0_ *+ E*_0_*\|treat* | 367 | 3 | **272** | **0** | 322 | 6 |
| #13 | *E =* (*k + k\|type*)*CU + E*_0_ *+ E*_0_*\|type* | 376 | 12 | 280 | 8 | 331 | 14 |
| #14 | *E =* (*k + k\|type*)*CU + E*_0_ *+ E*_0_*\|treat + E*_0_*\|type* | 385 | 21 | 292 | 20 | 338 | 21 |
| #15 | *E =* (*k + k\|type*)*CU + E*_0_ *+ E*_0_*\|*(*treat * type*) | 370 | 6 | 276 | 4 | 342 | 25 |
| #16 | *E =* (*k + k\|treat + k\|type*)*CU + E*_0_ | 368 | 4 | 277 | 5 | 321 | 4 |
| #17 | *E =* (*k + k\|treat + k\|type*)*CU + E*_0_ *+ E*_0_*\|treat* | 371 | 7 | 277 | 5 | 355 | 38 |
| #18 | *E =* (*k + k\|treat + k\|type*)*CU + E*_0_ *+ E*_0_*\|type* | 376 | 12 | 280 | 8 | 331 | 14 |
| #19 | *E =* (*k + k\|treat + k\|type*)*CU + E*_0_ *+ E*_0_*\|treat + E*_0_*\|type* | 372 | 8 | 276 | 4 | 327 | 10 |
| #20 | *E =* (*k + k\|treat + k\|type*)*CU + E*_0_ *+ E*_0_*\|*(*treat * type*) | 372 | 8 | 280 | 8 | 342 | 25 |
| #21 | *E =* (*k + k\|*(*treat * type*))*CU + E*_0_ | 368 | 4 | 277 | 5 | 321 | 4 |
| #22 | *E =* (*k + k\|*(*treat * type*))*CU + E*_0_ *+ E*_0_*\|treat* | 377 | 13 | 285 | 12 | 330 | 13 |
| #23 | *E =* (*k + k\|*(*treat * type*))*CU + E*_0_ *+ E*_0_*\|type* | 374 | 10 | 280 | 8 | 346 | 30 |
| #24 | *E =* (*k + k\|*(*treat * type*))*CU + E*_0_ *+ E*_0_*\|treat + E*_0_*\|type* | 372 | 8 | 276 | 4 | 327 | 10 |
| #25 | *E =* (*k + k\|*(*treat * type*))*CU + E*_0_ *+ E*_0_*\|*(*treat * type*) | 372 | 9 | 281 | 8 | 325 | 9 |

| 5-HT_2A_ blockades | | monkey KY | | monkey MP | | monkey ST | |
| --- | --- | --- | --- | --- | --- | --- | --- |
|  | Model | BIC | ΔBIC | BIC | ΔBIC | BIC | ΔBIC |
| #1 | *E = k CU + E*_0_ | **428** | **0** | 540 | 24 | 401 | 4 |
| #2 | *E = k CU + E*_0_ *+ E*_0_*\|treat* | 445 | 17 | 526 | 9 | 406 | 8 |
| #3 | *E = k CU + E*_0_ *+ E*_0_*\|type* | 447 | 19 | 530 | 13 | 408 | 10 |
| #4 | *E = k CU + E*_0_ *+ E*_0_*\|treat + E*_0_*\|type* | 447 | 18 | 530 | 14 | 409 | 11 |
| #5 | *E = k CU + E*_0_ *+ E*_0_*\|*(*treat * type*) | 437 | 9 | 528 | 12 | 400 | 2 |
| #6 | *E =* (*k + k\|treat*)*CU + E*_0_ | 435 | 6 | 545 | 29 | 403 | 5 |
| #7 | *E =* (*k + k\|treat*)*CU + E*_0_ *+ E*_0_*\|treat* | 443 | 15 | 555 | 38 | 412 | 14 |
| #8 | *E =* (*k + k\|treat*)*CU + E*_0_ *+ E*_0_*\|type* | 436 | 8 | **516** | **0** | **398** | **0** |
| #9 | *E =* (*k + k\|treat*)*CU + E*_0_ *+ E*_0_*\|treat + E*_0_*\|type* | 448 | 19 | 542 | 25 | 409 | 11 |
| #10 | *E =* (*k + k\|treat*)*CU + E*_0_ *+ E*_0_*\|*(*treat * type*) | 439 | 10 | 533 | 16 | 401 | 3 |
| #11 | *E =* (*k + k\|type*)*CU + E*_0_ | 438 | 9 | 521 | 5 | 403 | 5 |
| #12 | *E =* (*k + k\|type*)*CU + E*_0_ *+ E*_0_*\|treat* | 435 | 7 | 519 | 3 | 399 | 1 |
| #13 | *E =* (*k + k\|type*)*CU + E*_0_ *+ E*_0_*\|type* | 444 | 16 | 528 | 12 | 405 | 7 |
| #14 | *E =* (*k + k\|type*)*CU + E*_0_ *+ E*_0_*\|treat + E*_0_*\|type* | 456 | 27 | 539 | 23 | 417 | 19 |
| #15 | *E =* (*k + k\|type*)*CU + E*_0_ *+ E*_0_*\|*(*treat * type*) | 436 | 8 | 531 | 15 | 398 | 0 |
| #16 | *E =* (*k + k\|treat + k\|type*)*CU + E*_0_ | 438 | 10 | 521 | 4 | 402 | 4 |
| #17 | *E =* (*k + k\|treat + k\|type*)*CU + E*_0_ *+ E*_0_*\|treat* | 434 | 6 | 545 | 29 | 404 | 6 |
| #18 | *E =* (*k + k\|treat + k\|type*)*CU + E*_0_ *+ E*_0_*\|type* | 443 | 15 | 529 | 12 | 405 | 7 |
| #19 | *E =* (*k + k\|treat + k\|type*)*CU + E*_0_ *+ E*_0_*\|treat + E*_0_*\|type* | 438 | 10 | 524 | 8 | 403 | 5 |
| #20 | *E =* (*k + k\|treat + k\|type*)*CU + E*_0_ *+ E*_0_*\|*(*treat * type*) | 439 | 11 | 532 | 16 | 400 | 2 |
| #21 | *E =* (*k + k\|*(*treat * type*))*CU + E*_0_ | 440 | 11 | 521 | 5 | 402 | 4 |
| #22 | *E =* (*k + k\|*(*treat * type*))*CU + E*_0_ *+ E*_0_*\|treat* | 447 | 19 | 530 | 14 | 410 | 12 |
| #23 | *E =* (*k + k\|*(*treat * type*))*CU + E*_0_ *+ E*_0_*\|type* | 439 | 11 | 536 | 19 | 402 | 4 |
| #24 | *E =* (*k + k\|*(*treat * type*))*CU + E*_0_ *+ E*_0_*\|treat + E*_0_*\|type* | 440 | 11 | 524 | 8 | 401 | 3 |
| #25 | *E =* (*k + k\|*(*treat * type*))*CU + E*_0_ *+ E*_0_*\|*(*treat * type*) | 443 | 15 | 525 | 9 | 406 | 8 |

| 5-HT_4_ blockades | | monkey KY | | monkey MP | | monkey ST | |
| --- | --- | --- | --- | --- | --- | --- | --- |
|  | Model | BIC | ΔBIC | BIC | ΔBIC | BIC | ΔBIC |
| #1 | *E = k CU + E*_0_ | 445 | 5 | 307 | 2 | 336 | 8 |
| #2 | *E = k CU + E*_0_ *+ E*_0_*\|treat* | 449 | 9 | 313 | 9 | 336 | 7 |
| #3 | *E = k CU + E*_0_ *+ E*_0_*\|type* | 453 | 14 | 318 | 13 | 338 | 10 |
| #4 | *E = k CU + E*_0_ *+ E*_0_*\|treat + E*_0_*\|type* | 453 | 14 | 318 | 13 | 340 | 11 |
| #5 | *E = k CU + E*_0_ *+ E*_0_*\|*(*treat * type*) | 444 | 4 | 309 | 4 | 331 | 2 |
| #6 | *E =* (*k + k\|treat*)*CU + E*_0_ | 451 | 11 | 313 | 8 | 341 | 12 |
| #7 | *E =* (*k + k\|treat*)*CU + E*_0_ *+ E*_0_*\|treat* | 460 | 20 | 321 | 17 | 349 | 21 |
| #8 | *E =* (*k + k\|treat*)*CU + E*_0_ *+ E*_0_*\|type* | **440** | **0** | **305** | **0** | **328** | **0** |
| #9 | *E =* (*k + k\|treat*)*CU + E*_0_ *+ E*_0_*\|treat + E*_0_*\|type* | 457 | 18 | 321 | 17 | 341 | 13 |
| #10 | *E =* (*k + k\|treat*)*CU + E*_0_ *+ E*_0_*\|*(*treat * type*) | 448 | 9 | 313 | 8 | 335 | 6 |
| #11 | *E =* (*k + k\|type*)*CU + E*_0_ | 444 | 5 | 309 | 4 | 333 | 4 |
| #12 | *E =* (*k + k\|type*)*CU + E*_0_ *+ E*_0_*\|treat* | 443 | 4 | 307 | 2 | 329 | 1 |
| #13 | *E =* (*k + k\|type*)*CU + E*_0_ *+ E*_0_*\|type* | 452 | 13 | 316 | 11 | 337 | 9 |
| #14 | *E =* (*k + k\|type*)*CU + E*_0_ *+ E*_0_*\|treat + E*_0_*\|type* | 462 | 23 | 326 | 21 | 347 | 18 |
| #15 | *E =* (*k + k\|type*)*CU + E*_0_ *+ E*_0_*\|*(*treat * type*) | 447 | 7 | 311 | 6 | 333 | 5 |
| #16 | *E =* (*k + k\|treat + k\|type*)*CU + E*_0_ | 444 | 5 | 309 | 4 | 331 | 2 |
| #17 | *E =* (*k + k\|treat + k\|type*)*CU + E*_0_ *+ E*_0_*\|treat* | 451 | 11 | 313 | 8 | 342 | 13 |
| #18 | *E =* (*k + k\|treat + k\|type*)*CU + E*_0_ *+ E*_0_*\|type* | 452 | 13 | 316 | 11 | 337 | 8 |
| #19 | *E =* (*k + k\|treat + k\|type*)*CU + E*_0_ *+ E*_0_*\|treat + E*_0_*\|type* | 448 | 8 | 311 | 7 | 333 | 5 |
| #20 | *E =* (*k + k\|treat + k\|type*)*CU + E*_0_ *+ E*_0_*\|*(*treat * type*) | 448 | 9 | 313 | 8 | 333 | 5 |
| #21 | *E =* (*k + k\|*(*treat * type*))*CU + E*_0_ | 444 | 5 | 309 | 4 | 333 | 4 |
| #22 | *E =* (*k + k\|*(*treat * type*))*CU + E*_0_ *+ E*_0_*\|treat* | 453 | 14 | 317 | 13 | 339 | 11 |
| #23 | *E =* (*k + k\|*(*treat * type*))*CU + E*_0_ *+ E*_0_*\|type* | 451 | 12 | 315 | 11 | 337 | 8 |
| #24 | *E =* (*k + k\|*(*treat * type*))*CU + E*_0_ *+ E*_0_*\|treat + E*_0_*\|type* | 448 | 8 | 311 | 7 | 332 | 4 |
| #25 | *E =* (*k + k\|*(*treat * type*))*CU + E*_0_ *+ E*_0_*\|*(*treat * type*) | 449 | 9 | 313 | 8 | 335 | 7 |

*CU* and *E*_0_ indicate the remaining cost and the intercept, respectively. *k*(*treat*), *k*(*type*)*,* *e*(*treat*)*,* and *e*(*type*) denote the random effects of blocking 5-HT on parameters *k* and *e*, respectively. The probability distribution of the random effects were as follows; *k_treat_* and *k_type_* were ~N(0,σ^2^_treat_) and ~N(0,σ^2^_type_), respectively. (*k’_treat_, e’_treat_*) and (*k’_type_, e’_type_*) were ~biNorm(0, ∑_treat_) and ~biNorm(0, ∑_type_), respectively. *E*, error rate; *treat*, treatment condition (antagonist or control): *type*, trial type (delay or work). BIC is a relative measure of quality for the models (#1-25). ΔBIC denotes the difference from the minimum BIC.
