# Supplementary material for: Reduced serotonergic transmission alters sensitivity to cost and reward via 5-HT1A and 5-HT1B receptors in monkeys
Source: PLoS Biol. 2024 Jan 1;22(1):e3002445. doi: 10.1371/journal.pbio.3002445 (PMC10758260; doi:10.1371/journal.pbio.3002445)
Supplement: Attachment — Submitted filename: ResponseToReviewers1002.docx [file pbio.3002445.s017.docx]

We are grateful to the three reviewers for their invaluable contributions, including both their positive assessments and their invaluable insights, which have greatly improved the quality of our work. Before going into a point-by-point response to the reviewers' concerns, we want to note that we have adjusted our conclusions based on the results we observed after refining the model-based analysis, as suggested by reviewer #3. Specifically, the analysis now leads us to conclude that 5-HT_1A_R and 5-HT_1B_R contribute differentially to incentive- and cost-related aspects of motivation, but without a double dissociation. Despite this change, our study still provides important insights into the seemingly complex relationship between 5-HT deficits and reduced motivation—5-HT affects cost and incentive processing through different mechanisms.

REVIEWERS' COMMENTS:
Reviewer #1:

The serotonin (5-HT) system is believed to be a significant factor in depression and other psychiatric disorders that involve decreased motivation. However, the precise role of 5-HT in motivation remains unclear. In this study, researchers manipulated the 5-HT system in monkeys using drugs and gauged the effects on motivation in a goal-directed task. They discovered that inhibiting 5-HT synthesis reduced motivation, which could be separated into value-dependent and value-independent components. The researchers also employed specific drugs to block different 5-HT receptor subtypes and found that blocking 5-HT1A receptors reduced motivation in a value-independent manner, whereas blocking only 5-HT1B receptors lessened the impact of incentive value on motivation. These results imply that the 5-HT system has a complex role in motivation, with various receptor subtypes mediating distinct processes that lead to reduced motivation in 5-HT deficiency. Although the experimental procedure is commendable, there are some critical concerns and limitations. We think that the authors can address these after revising the manuscript. 

R: We thank the reviewer for this positive assessment of our manuscript.

Major points:

(1)	How to relate "refusal rate" with motivation. 

(1-1)	The paper's definition of motivation appears to be ambiguous, and its interpretation of cost, reward, and motivation is the main weakness. The authors used "refusal rate" as a measure of motivation, which is closely linked to the Go-Nogo movement process and may not fully capture the complexity of motivational processing. 

R: We thank the reviewer for raising this important issue regarding the definition and interpretation of motivation in our study. We have clarified the relationship among cost, reward, and motivation in the Introduction and have made it consistent with the interpretation of our results throughout the manuscript by mapping our findings onto an incentive/cost model of motivation.

In addition, after careful consideration, we acknowledge that the term "refusal rate" is an interpretation rather than a direct measure of motivation, and this may have caused confusion and made the text unclear. In light of this, we have decided to replace the term "refusal" with "error" throughout the manuscript. We believe that this is a better and more straightforward representation of the nature of our measurements.

We would like to clarify that the two tasks used in our study did not require the monkeys to make explicit decisions about whether or not to perform a trial. Instead, the error rates reflect the monkeys’ lack of motivation to complete the task and obtain the rewards. In the course of previous studies using similar tasks, we have consistently shown that error rates in these tasks can be modeled using independent variables such as subjective reward value (e.g., Minamimoto et al., JNP 2009; Simmons et al., JNS 2010; Bouret et al, JNS 2010; Nagai et al., 2016; Fujimoto et al., JNS 2019, and others), cost-based discounting of the reward value (Minamimoto et al., PLOS One 2012; Hori et al., eLife 2021), and impact of internal driver such as satiation (Minamimoto et al., Exp Brain Res 2012; Fujimoto et al., JNS 2019).

We also would like to emphasize that these previous studies provide evidence not only for the correlation between behaviors and motivation, but also for the neural correlates and causality of motivation. In addition, reaction time - another behavioral measure often considered to reflect motivational processes in parallel with incentive - tends to only partially reflect these variables, making modeling impossible. Therefore, we believe that our tasks and error measures quantitatively capture the complexity of motivational processing, specifically how the brain processes these variables to control motivation.

In response to the reviewer's comments, we have revised the Results section to emphasize and clarify these points. We believe that these revisions address your concerns and improve the overall clarity of our manuscript.

(1-2)	Moreover, the current definition of "Refusal rate" encompasses various types of errors, including early release before the green target appeared and late release after the green target. It would be beneficial for the authors to separate these errors based on their occurrence before or after the green target and present the results separately. These errors may have different underlying meanings, with early releases possibly indicating impulsivity and late releases possibly indicating decreased attention. Additionally, the monkeys' release of the bar to indicate their cost evaluation may be prone to errors, such as releasing the bar even if they want to continue the trial or not releasing the bar if they are not paying enough attention to the task. 

R: We appreciate the reviewer's insightful comment about the need to separate errors based on whether they occur before or after the green target as they may have different underlying mechanisms.

We would like to explain our conceptual reason for integrating these errors as a single measure in this and previous studies. First, because the basic task requirement (i.e., releasing the bar after go signal) does not involve explicit decisions about whether to perform a trial or not and is very easy for the monkeys to do, any type of erroneous performance would reflect the insufficient motivation of the monkeys to complete the task and obtain the expected rewards. As noted in the response to the previous comment, error rates can be modeled by expected reward value and satiation level, consistent with the concept of incentive motivation. Second, even when we treated the early and late errors separately, an inverse relationship between reward size and the proportions of errors was observed for both early and late errors (Minamimoto et al., JNP 2009, Fig. S1), suggesting that both are similarly affected by incentive values under normal conditions. Given that the proportions of these errors vary between monkeys, it makes more sense to treat them together. In addition, to maintain consistency with previous literature, including studies examining the effects of dopamine antagonists (Hori et al., PlosBiol 2021), we use error rates as the central behavioral measure throughout this study.

Nonetheless, we fully acknowledge the importance of analyzing early and late errors separately, as they can provide valuable insight into impulsivity and attentional lapses, respectively. In response to the reviewer's suggestion, we have provided a new plot showing these errors separately (Figure 2C). Again, there appears to be an inverse relationship between reward size and the proportion of early and late errors in the control condition. Notably, administrating pCPA increased both early and late errors, with late errors tending to increase more. Together with the analysis of the early error ratio in Figure 2D (previously Figure 2C) and the accompanying statistical analysis, these updated figures allow for the separate examination of these two types of errors (Figure 2C). We have modified the Results section accordingly.

*"Administering pCPA increased both early and late errors across all reward-size conditions (Fig 2C), with late errors tending to increase more (e.g., Fig 2D; ..." (in Results; lines 141–143)*

In addition, as the reviewer pointed out, we acknowledge that there are a variety of mechanisms behind errors, including what the reviewer mentioned as not necessarily relevant to value or cost, which were classified in this study as the errors orthogonal to incentive values (i.e., parameter e). We mentioned this in the Discussion as follows:

*"Although errors can occur through a variety of mechanisms such as impulsivity and decreased attention, our results suggest that the incentive-independent changes after blockade may be related to an overestimation of the impending cost" (in Discussion; lines 344–347)*

(1-3)	While the mathematical modeling of the refusal rate is of high quality and meaningful, it is challenging to fully credit it as a measure of motivational processing. Therefore, it is essential for the authors to clearly dissociate the various factors contributing to refusal rate, including types of movement (i.e., go-nogo, preparation and/or initiation of movement), cost, motivation, and error. If the authors cannot separate these factors, they should at least acknowledge their limitations in the discussion section.

R: We appreciate the reviewer’s comments regarding the various factors contributing to the error rate and their potential impact on quantifying motivation. In response to the concerns described above, we have made an amendment by replacing the term "refusal" with "error" in our manuscript. This modification allows us to provide a clearer understanding of the relationship between reward, cost, and error, which aligns with the incentive value theory of motivation. Our intention is to ensure that motivational processes are adequately captured and interpreted quantitatively.

Our findings suggest that the increase in error rate due to 5-HT depletion can be decomposed into two distinct components. One component can be explained by a decrease in incentive and the other component (i.e., increase in parameter e) orthogonal to incentive, which our experiments indicate is the cost. Indeed, the second component was also exclusively replicated by 5-HT_1A_R blockade, and was related to increased cost sensitivity.

We acknowledge that the second component may also include factors beyond cost sensitivity, such as something related to preparation or initiation of bar movement, which we did not directly examine or control for in this study. It could also be related to aversion/punishment as previous 5-HT literature suggest. We include a discussion of the importance of addressing this in future studies.

*"Studies examining the activity of 5-HT neurons and their optogenetic manipulation also encourage us to consider motivation-independent factors beyond cost sensitivity. 5-HT neurons convey information not only about reward but also about aversive events [58, 59]. In addition, another example of complexity is the involvement of 5-HT in orchestrating behavioral inhibition [60]. Future investigation will be needed to examine the consequence of manipulating 5-HT transmission in nonhuman primates in the context of reward and punishment." (in Discussion; lines 405–410)*

(2)	The experimental results in this paper require clearer descriptions.

(2-1)	 In Fig. 1E, it would be helpful to include information about the number of sessions per animal. It appears that the authors used data from 11 sessions for each condition (control, day 1, and day 2), obtained from 4 monkeys. Knowing which monkey contributed to each session would be beneficial.

R: In response this, and similar comments from reviewer 3, we have made a supplementary table (S2 Table) that provides detailed information of the operations and measurements performed on each monkey. To ensure clarity and transparency, we have also updated the legend for Fig. 1E in the Results section.

(2-2)	Regarding Fig. 4, it is noteworthy that 5HT4R also increased parameter "a." The authors could provide their opinion on this result to help readers understand its significance. Additionally, statistical analysis could be performed for Fig. E and F to support the findings.

R: Thank you for your valuable comments. The current study focuses on how 5-HT depletion leads to decreased motivation. Because the effect of 5-HT_4_R blockade did not mimic the increase in error rate observed with 5-HT depletion, we feel that focusing on it too much might be a distraction from the main topic. However, given that being able to increase motivation via 5-HT_4_R blockade is certainly important, especially in terms of a potential drug target for treating loss of motivation, we briefly include our opinion of this finding in the discussion.

*"Similarly, the current study showed that blockade of 5-HT_4_R significantly increased motivation, suggesting that normal 5-HT transition via 5-HT_4_R decreases incentive (Fig. 4E). Importantly, this implies that 5-HT_4_R is a potential target for treating loss of motivation. Our results, together with other studies, indicate that different 5-HTR subtypes likely modulate motivation in different directions, suggesting a more complicated view of what SSRIs do in humans, as they are assumed to enhance 5-HT transmission equally at multiple receptor subtypes." (in Discussion; lines 398–403)*

Regarding the comments on the statistical analysis, the LMM analysis allowed us to identify which factors were significantly affected by treatment, as indicated by model selection with BIC. We now report this explicitly in the Results section as follows:

*"Blocking 5-HT_4_R consistently decreased error rates (Fig. 4D) and significantly increased a to approximately 150% (Fig. 4E; Model #30, S5 Table). Thus, it is unlikely that 5-HT_2A_R or 5-HT_4_R directly mediates the behavioral changes observed with 5-HT depletion. " (in Results; lines 191–193)*

(2-3)	In Fig. 5D, it would be useful to perform statistical analysis to demonstrate the significance of 5HT1A.

R: We thank the reviewer for pointing this out. As mentioned above, the LMM analysis was able to identify which factor(s) were significantly affected by treatment. We now report this explicitly in the Results section.

*"Blockade of 5-HT_1A_R significantly increased delay discounting in two of three monkeys and workload discounting in all three cases, whereas it significantly increased cost-independent error in only one monkey (Fig 5D)." (in Results; lines 247–249)*

(2-4)	Finally, in the introduction section, it would be beneficial to explain more about why incentives and costs are important in motivation and 5HT systems. Currently, these concepts appear suddenly and may be difficult to understand in relation to the 5HT systems.

R: We appreciate the reviewers' valuable advice. As the reviewer pointed out, the relationship between incentive, cost, and motivation was not clear in the original manuscript. We have revised the Introduction to provide a clearer concept of these relationships and how they relate to 5-HT systems, as follows.

*"* *The motivation to do any voluntary goal-directed behavior is a trade-off between incentive and the expected cost. The subjective value of the reward expected upon achieving the goal determines the incentive (or incentive value), which has a positive influence on motivation. The expected cost is a subjective valuation of what must be paid to earn the reward (e.g., waiting, taking a risk, or putting forth effort) and has a negative influence [1-3]." (in Introduction; lines 20–24)*

*"5-HT deficiency is currently thought to reduce incentive (e.g., blunting/eliminating pleasure from rewards) and/or increase the expected cost (e.g., waiting becomes intolerable, resulting in impulsivity) [4, 5]." (in Introduction; lines 28–30)*

Minor points
1.	Did the administration of pCPA have any effects on the monkeys' behavior outside of task performance, such as changes in their behavior in their cages? If so, it would be helpful to provide some examples of these observations in the text.

R: To assess the effect of pCPA administration on behavior outside the task context, we examined the amount of total activity exhibited by the monkeys in their home cage. We observed that the amount of behavior displayed by the monkeys decreased in the days following pCPA treatment (day 1 and day 2). We have included these results in the Results section and modified the Methods section, accordingly.

*"In addition to the slower responses, pCPA treatment also significantly decreased general motor activity in the home cage (4 sessions in 3 monkeys; one-way ANOVA, main effect of treatment, F_(1,11)_ = 7.28, p = 0.021)" (in Results; lines 138–140)*

*"Total motor activity in the home cage was also analyzed after the behavioral test until room lights were turned off each day (16:00 - 21:00 h) after the control and post-pCPA sessions. Motor activity was recorded using motion-sensitive accelerometers (ActiCal, Motion Biosensors, PHILIPS) attached to the monkeys’ collars." (in Methods; lines 480–483)*

2.	Regarding Fig. 3, in addition to the distribution of 5HT receptor subtypes in the brain, could you also provide information about their total amount in the brain and body?

R: The data presented in Figure 3 using PET imaging are derived from the measurement of PB_ND_, that is, tracer binding relative to non-specific binding measured in a reference region (in this case, the cerebellum). PET imaging does not provide a direct measure of absolute amounts of receptor distribution or density throughout the brain or body. So, unfortunately, we cannot provide that information. To clarify this point, we have revised the Methods section to include an explanation of what the BP maps provide.

*"Because BP_ND_ is assumed to linearly reflect the spatial distribution and availability of receptors throughout the brain region, we rescaled each BP map by its minimum and maximum values to allow comparison of relative receptor distribution." (in Methods; lines 540–542)*

3.	On page 12, line 273, it is mentioned "Therefore, blockade of 5HT1BR". Is this supposed to be "5HT1AR" instead?

R: Thank you for pointing this out. 5-HT_1B_R is correct, but we realize that this was not well-written. Therefore, we revised it as follows:

*“In contrast, blockade of 5-HT_1B_R significantly decreased delay discounting in only one of three cases.” (in Results; lines 249–250)*

4.	The study used male monkeys exclusively. Could you discuss whether this could potentially affect the results and their interpretation?

R: We appreciate the reviewer’s suggestion to discuss the potential impact of sex on the results and their interpretation. To address this limitation and to acknowledge the potential influence of sex on 5-HT motivational function in primates, we have included the following limitation statement in the Discussion section:

*"Third, it should be noted that the present study was conducted only in male monkeys. Given that sex differences in 5-HT function are known [62], future studies should examine whether the present results are applicable to female monkeys." (in Discussion; lines 429­­–431)*

5.	In Figure 4A, there appears to be a significant variation among the monkeys. Could you discuss this observation in more detail in the discussion section?

R: As the reviewer suggested, we included the following discussion regarding the variation among the monkeys:

*"5-HT_1A_R blockade induced increases in error rate during the reward-size task, with individual variation—the increase was high in two monkeys and relatively small in another (Fig. 4). Despite the quantitative differences, these effects were qualitatively consistent across individuals in the sense that they could be decomposed into two factors: lower incentive and another factor orthogonal to incentive values. Because one monkey exhibited relatively weaker effects for both factors, the variation may be due to pharmacological effects, including differences in 5-HT_1A_R expression level and/or antagonist pharmacokinetics." (in Discussion; lines 386­­–392)*

6.	Could you consider the possibility that 5HT1B occupancy in the occipital cortex may have affected the results, potentially influencing the visual system and causing illusions? This could be discussed in the limitations or future directions section of the paper.

R: We appreciate the reviewer's suggestion and consideration of potential effects on the visual system due to 5HT_1B_R receptor occupancy. 5-HT_1B_ and 5-HT_2A_ receptors are known to be abundant in the visual cortex of primates, including macaques (Watakabe et al., CerCortex 2009). The 5HT_1B_R is known to be involved in contrast-based edge and contour detection in the retinal image, potentially affecting the visual processing system (Shimegi et al., JP, Paris 2016); therefore, it is possible that blockade of 5HT_1B_R modulated visual sensitivity or perception.

However, we believe that the effects on the visual system were unlikely in our study for the following two reasons. First, since the potential effect on visual perception would be contrast sensitive, the use of high-contrast images in our task (Figs. 1A and 5C) should not confound the results. Second, even if 5-HT_1B_R blockade were to affect task performance, changes in the detection of visual signals would uniformly influence the trial types, which contradicts what we observed (i.e., changing error rate depending on reward size).

We have included the following discussion regarding the potential effects of 5HT_1B_R blockade on visual processing and perception in the Discussion section.

*"Primate visual cortex is rich in 5-HT_1B_R [29], which has been implicated in contrast-based edge/contour detection [30], a potential mediator of the observed behavioral changes. However, the use of high-contrast stimuli (i.e., white/black cues and red/green signals) may prevent potential effects due to contrast sensitivity. In fact, the behavioral effects of 5-HT_1B_R blockade on the current study were reward-size dependent, which is contrary to what would happen if it was related to changes in the visual system; analyzing data by reward size would result in uniform effects, not reward-size dependent ones." (in Discussion; lines 322–328)*

Reviewer #2:

This was an interesting manuscript, which reported on the role of specific 5-HT receptor subtypes in aspects of motivation. The experiments were well designed and well conducted. My specific comments, which are intended to help the authors with the presentation of their results, are listed below.

R: We thank the reviewer for this positive evaluation and their valuable suggestions.

Major Comments:

The authors have a short paragraph in the discussion mentioning some of the rodent studies focusing on the effects of SSRIs on effort based choice and motivation, as well as one human paper. Although the authors state that "A rodent study" involving SSRIs showed effects on cost based decision making, a more recent paper also showed the same type of effects, including tests of both lever pressing and wheel running (Presby et al. 2021). Moreover, selection of this one human paper is somewhat limited, because the authors do not cite the multiple papers from the clinical literature showing that SSRIs are relatively poor at treating motivational dysfunction in depressed people (Papakostas et al. 2006; Fava et al. 2014; Cooper et al. 2014; Rothschild et al. 2014; Popovic et al. 2015), and can even induce fatigue as an adverse event in some people. Since the introduction and discussion of this manuscript focus extensively on the role of the 5-HT in depression and the effects of
SSRIs, especially motivational aspects, the references cited above are relevant for showing the conflicting results about the role of 5-HT in motivational dysfunction in depression. The human literature itself is complicated, so the findings do not seem to be simply understood in terms of a rodent vs. primate difference.

R: We appreciate the reviewer's valuable feedback regarding the inclusion of additional relevant literature in our manuscript. In response to this, we now refer to the suggested papers and highlight the conflicting results and the complex nature of the findings in both rodents and humans. We believe that this will provide a more balanced perspective on the role of 5-HT in motivational dysfunction and the effects of SSRIs in the clinical context.

*"* *A rodent study of cost-benefit trade-offs showed that SSRIs reduced effort expenditure [43]. Another study also reported similar effects of SSRIs, including suppression of higher effort activities such as lever pressing and wheel running [44]. Other rodent studies have shown that 5-HT depletion with pCPA had no effect on the tendency of rats to make cost-based choices [7, 45]. One human study showed that patients taking SSRIs produced more effort for monetary incentives, which was mediated by a reduction in the cost of effort, but no change in the weights of incentives [5]. In contrast, several human studies have reported that SSRIs are relatively ineffective in treating motivational dysfunctions such as fatigue and anergia [44, 46-50].” (in Discussion; lines 367–375)*

In the discussion, the authors discuss their results showing that no other 5-HT receptors were involved in effort-related aspects of motivation, and cite one rodent study involving 5-HT 2A receptors. However, Bailey et al. (2018) showed that 5-HT 2C antagonism enhanced effort-based motivation in rats. Interestingly, this points to the involvement of a 5-HT receptor in mediating decreases in motivation rather than increases. Thus, it is possible that different 5-HT receptor subtypes are pushing motivational function in different directions.  This finding also suggests a more complicated view of what SSRIs are doing in humans, since they enhance 5-HT transmission at multiple receptor subtypes via blockade of 5-HT transport. 

R: We appreciate the reviewer's insightful comment regarding the potential involvement of different 5-HT receptor subtypes in mediating decreases or increases in motivation. Our study focused on investigating the specific receptors mediating the decrease in motivation that occurs with low 5-HT transmission. However, we recognize that other receptor subtypes are involved in mediating increases in motivation, which is an important aspect to consider, particularly in the context of SSRIs and their mechanism of action in humans.

Although not directly addressed in our study, the modulation of motivational function by receptor stimulation is a relevant and closely related issue. Moreover, because we found that blocking 5-HT_4_R seems to increased incentive despite inducing low 5-HT transmission, interpreting this finding in this context is also fruitful. We therefore have included this in the discussion and provide a more comprehensive view of the serotonergic system and its impact on motivation, specifically the potential role of different 5-HT receptor subtypes in pushing motivation in different directions as follows:

*"Another rodent study showed that antagonism of 5-HT_2C_R enhanced effort-based motivation [42], suggesting that it mediates decreases rather than increases in motivation. Similarly, the current study showed that blockade of 5-HT_4_R significantly increased motivation, suggesting that normal 5-HT transition via 5-HT_4_R decreases incentive (Fig. 4E). Importantly, this implies that 5-HT_4_R is a potential target for treating loss of motivation. Our results, together with other studies, indicate that different 5-HTR subtypes likely modulate motivation in different directions, suggesting a more complicated view of what SSRIs do in humans, as they are assumed to enhance 5-HT transmission equally at multiple receptor subtypes." (in Discussion; lines 396–403)*

The results demonstrated dissociations between different aspects of motivation. This should be a central focus of the discussion, because there is a tendency to use general terms such as "anhedonia" to subsume all aspects of motivational impairments into one broad construct. The present pattern of results emphasizes the dissociable nature of different aspects of motivation. 

R: We appreciate the reviewer's insightful comments. As we note at the top of this letter, the new analysis no longer supports the double dissociation of two receptor subtypes. However, our results still indicate that different aspects of motivation are governed through separate circuitry, and thus we fully agree that it is important to emphasize this point. As the reviewer points out, our findings underscore the need to avoid generalizing all motivational disorders with broad terms such as "anhedonia". Discussion of serotonin in the context of its relationship to multiple processes may also provide a more cautious view of motivational impairment, avoiding oversimplification of its multifaceted nature. In response to this comment, the following sentences are now included in the Discussion:

*"Our findings shed light on the mechanistic role of 5-HT in different aspects of motivational regulation and their relevance to different aspects of medications used to treat depression." (1st paragraph of Discussion, lines 269–271)*

*"We also emphasize that our results suggest that multiple factors and related brain processes may drive a single behavioral measure of motivation, namely, an increase in error rate. This may be important from a clinical perspective, as there is a tendency to use general terms such as “decreased motivation” or “anhedonia” to subsume all aspects of motivational impairment into one broad construct. Our results challenge this oversimplified view of motivational impairment and propose a more nuanced view in which multiple behavioral factors and neural processes underlie it." (in Discussion; lines 300–305)*

Reviewer #3:

In this article, the authors aim to dissect the contributions of different serotonin receptor subtypes to processing to cost and rewards. This is an important topic and a well-executed study. There is extensive evidence that serotonin plays an important role in motivation, yet the majority of studies have not addressed receptor specificity, which is particularly important in the case of serotonin, given the large nr of different receptors (relative to e.g. the dopamine system). This has potential clinical implications also given that reduced motivation for effortful tasks may arise through different cognitive and neurobiological mechanisms (e.g. in apathy, chronic fatigue, depression, all associated with serotonin). The authors completed a very impressive set of studies, combining global serotonin depletion (pCPA), PET localization of the different receptor subtypes, and drug administration, across a set of 2 tasks. 

That said, I have a number of concerns regarding primarily the framing and interpretation of the results, as well as a few more technical questions regarding the computational modelling.

R: We thank the reviewer for this positive assessment and for their constructive comments on our manuscript. We have addressed the reviewer's concerns and technical questions below.

The primary finding as the authors present this, is a double dissociation of the effects of 5HT1A vs 5HT1B blockade, where the former appears to affect the evaluation of costs, while the latter mediates the impact of incentive value on motivation. However, when evaluating the results, I find the effects of 5HT1A antagonism very convincing, but the effects of 5H1B blockade (and interpretation there-of) much less so (figure 4, figure 6). This is for a number of reasons: 

-	The 1B effects are much smaller, and appear to be drive by purely the lowest reward sizes. This may be because the base refusal rate (figure 4) is very low already (varies a little bit per monkey, but for most it has asymptoted by a reward size of 2-4. Thus, if 1B blockade lowers refusal rates, it only has a very small 'window' in which it can do this. 

R: We appreciate the reviewer's insightful comments regarding the observations after 5-HT_1B_R blockade. In this part of the experiment, our goal was to identify the receptor subtypes involved in the motivation reduction that we observed with 5-HT depletion. As a result, we expected to observe an increase in error rates rather than a decrease.

It is important to note that the measure of our task, error rate, is not only indicative of motivation, but can also be transformed into a measure of motivational value (Minamimoto et al., 2009). By modeling the fitting curve, we can infer a monkey’s ability to estimate reward values quantitively. We agree that the baseline error rates, as shown in Figure 4, appear to plateau at higher reward sizes. However, this actually indicates that the monkeys are performing normally based on the expected reward size and the constant cost. On the other hand, an upward shift with increasing parameter e, as seen in 5-HT_1A_R blockade, suggests deviations from normal motivational processing that rely solely on incentive (i.e., overestimation of baseline cost). Note that no downward shift is assumed here, which would result in a reduction of parameter *e* from the baseline (usually 0).

It should also be noted that our measurement results combined with the model analysis showed a significant increase in error rates after 5-HT_1B_R blockade. Moreover, we observed significant decrease in error rates after 5-HT_4_R blockade. These clearly indicates that our measurement provides a sufficient window to assess changes in motivation that result from changes in incentive (i.e., parameter a).

In the revised manuscript, we have tried to provide a comprehensive explanation of the tasks used and the concept behind using error rates as a measure of motivation.

-	This dissociation hinges on the model comparison where you separately fit a and e. How reliably can both a and e can be fitted, given the low refusal base rate and early asymptote (i.e. at low reward sizes already)? Please include parameter recovery analyses to show that these can indeed be recovered independently in the parameter regimes that you report here (see also Collins & Wilson, 2019 eLife 10 simple rules for computational modelling), particularly with the floor effects of the refusal rates.

R: First, we did fit a and e simultaneously in the LMM. This confusion may be due to our statement in the Methods section of our original manuscript, "Using the selected model, parameter *a* and intercept *e* were estimated individually. (lines 560-561)" was misleading. We apologize for the confusion.

We have changed the sentence to make it clear:

*"Using the selected model, parameter a and intercept e were estimated simultaneously within each subject". (in Methods; lines 616–617)*

To address the reviewer's concern directly, we performed a Bayesian inference analysis that indicated how reliable two parameters are in fitting the data. This is an alternative to parameter recovery analysis that often yields fluctuating results when the sample size is not large enough, as in this case.

The figure shows the parameter estimation of the data for 5-HT_1A_R blockade and control in monkey KN, assuming that the random effects for the slope (*a*) and intercept (*e*) are homogenous across treatment conditions and subjects, corresponding to the best model of the LMM analysis: err ~ x + (x | mk_cond).

The red and green dots represent the posterior distribution of the model parameters from an Hamiltonian Monte Carlo simulation (iter_warmup=2000, iter_sampling=4000, and chains=4) for 5-HT_1A_R blockade and control conditions, respectively. The red and green circles indicate the 95th percentile for each condition. In both conditions, the parameter obtained from the best-fit LMM model (+) are very close to the maximum *a posteriori* (MAP) estimate from the simulation (black dots), suggesting a high reliability of the parameter estimates. All R-hat values were below 1.02, indicating that this Bayesian simulation was statistically converged. All random effects were statistically significant as the 95% confidence intervals deviated from 0.

-	Furthermore, for the computational modelling model comparison, the authors use the AIC, and find evidence that the model with most parameters is also the winning model. Generally, AIC penalizes very 'lightly' for model complexity, thereby favouring more complex models. If you use the somewhat more conservative BIC, does the same model win? 

R: We appreciate the reviewer’s insightful comment regarding model comparison using AIC and the concern about its tendency to favor more complex models due to its slight penalty for model complexity. In response to this concern, we have re-evaluated the model comparison using the more conservative Bayesian Information Criterion (BIC), as suggested. Through this analysis, we found that in the majority of cases, both BIC and AIC converge to select the same or very similar models, reinforcing the robustness of our findings.

However, in the case of 5-HT_1A_R blockade within the reward-size task (as shown in Figure 4), we noticed a discrepancy where the BIC favored the model (#30) that includes significant effects on both parameters *a* and *e*, while the AIC favored one (#5) with only a significant effect on *e*. This discrepancy prompted us to carefully consider the point raised by the reviewer regarding AIC's tendency to penalize model complexity lightly. As a result, we decided to use BIC for all model selections throughout the study, recognizing its stricter stance on model complexity.

This analytical adjustment led to significant findings that required us to adjust our conclusions. Specifically, in contrast to the initial assertion in the paper regarding the independent involvement of 5-HT_1A_R and 5-HT_1B_R in different aspects of motivation, the analysis now leads us to conclude that the receptor subtypes contribute differentially to incentive- and cost-related aspects of motivation, but without a double dissociation. This refined understanding is consistent with the reviewer's comment that 5-HT_1A_R appears to be a more substantial contributor. We have therefore addressed this issue in a coherent manner and updated the paper accordingly (see below).

We also re-evaluated the analysis of the relationship between RT and error (Figure 2E). We recognized that the dataset may not have been large enough because two different models had comparable BIC values. Therefore, to increase the statistical robustness of our analysis, we split the data from each session into first and second halves, allowing us to increase the number of RT-error pairs under a given motivational condition (i.e., incentive × drive). This modified analysis yielded consistent results, confirming that the same model had been selected between AIC and BIC.

The conclusions did not change when BIC was used instead of AIC to analyze the work/delayed task data. In a small number of cases, the significance and the parameters were changed; Figure 5 and Figure S4 have been revised accordingly.

Even when the effects of 1B turn out to be indeed robust, the authors should highlight the vast difference in effect size, and tone down the double dissociation nature of the conclusions, but rather focus on the 1A effects. 

R: Thank you very much for the valuable comment. As the reviewer pointed out, the impact of 5-HT_1A_R antagonism was higher than that of 5-HT_1B_R. Indeed, being able to see this is one of the advantages of directly comparing different receptor subtypes at a similar occupancy. Thus, we emphasize this in the Discussion as follows:

*"* *The incentive-dependent component of decreased motivation was reproduced by both 5-HT_1A_R and 5-HT_1B_R blockade, with the latter showing a relatively stronger effect. Blockade of 5-HT_1A_R produced both value-dependent and -independent decreases in motivation, highlighting a central role in decreased motivation." (in Discussion; lines 309–312)*

The dissociation of components related to decreased motivation originally observed after the 5-HT depletion experiments serves as a fundamental and generalizable indication of impairment within the 5-HT system. Our subsequent investigation aimed to elucidate the specific involvement of different 5-HT receptor subtypes, leading us to identify 5-HT_1A_R and 5-HT_1B_R as potential contributors.

Despite the greater effect of 5-HT_1A_R blockade, it is important to acknowledge the significant role played by 5-HT_1B_R. Notably, the depletion experiments also showed a comparable effect, namely a reduction in incentive (parameter a). Therefore, we believe it is critical to emphasize the dissociable nature of the factors affecting motivation (actually, suggested by reviewer#2). We also have emphasized how our findings map onto the incentive/cost model of motivation, as suggested by reviewer #1.

We have carefully addressed this issue in the revised version of the manuscript, providing a thorough discussion to ensure a balanced and comprehensive interpretation of our results and their implications.

MINOR POINTS

Please include colour legends in all figures.  
In the figures with reward size, please also include the '0': I assume the lowest reward size is 1?

R: As suggested by the reviewer, we have added colored legends to all the figures and labeled 0 for reward size.

The pCPA study shows increasing refusal rates over the course of testing. Did you also collect a control sample that just completed 3 testing sessions, as this effect may simply be a function of time / nr of sessions completd, rather than pCPA. If no control group was included, please make sure to highlight this caveat. 

R: Thank you for your thoughtful comment. Unfortunately, the pCPA study did not include a non-pCPA control injection. We acknowledge that this omission could potentially introduce confounding factors as the reviewer pointed out. To address this concern, we performed an additional analysis using the behavioral data collected after the saline injection and following first and second days in the 5-HT_1A_R blockade experiment, confirming no effect of days without pCPA. We have mentioned this control in the Methods section of the revised manuscript.

*"* *Although we did not perform a control test in the same cohort, we looked at the saline injection data and confirmed that the act of injection itself did not affect error rates in the two days after injection [two-way ANOVA, main effect of days, F_(2, 138)_ = 2.3, p = 0.10]. " (in Methods; lines 477–480)*

Figure 1A: describe the task in more detail in the caption. 

R: As suggested, we included the details of the task in the legend of Figure 1 as follows:

*"(C) Reward-size task. Left: Sequence of events during one trial. A monkey initiated a trial by touching the bar in the chair. ... After each correct trial, a new cue reward-size pair was picked from the set of four at random. Right: Relationship between visual cues and reward size."*

Figure 1C: the term 'early release rate' from the caption is much more informative than 'error rate' - I would recommend putting that in the figure also. 

R: Following the reviewer's suggestion, we replaced "early error ratio" with "early release rate" in Figure 2C (Figure 2D in revision). (Note that we did not find the term early release rate in Figure 1C, so we assumed it was Figure 2C, but please point out if this is not the case).

Figure 5: which monkeys are these plots referring to? All only in monkey ST? 

R: Yes, all plots include the data collected form monkey ST, as a representative case. We indicate this in the figure and the legend.

Can you add a discussion as to what you believe the effects of the different drugs, particularly the 5HT1A and 1B antagonis, have on overall serotonin signalling? 

R: Thank you for this suggestion. We now include the following discussion as a limitation in the revised manuscript.

*"Finally, because 5-HT_1A_R and 5-HT_1B_R are located in the cell soma and presynaptic terminals of 5-HT neurons and serve as autoreceptors that regulate 5-HT neuronal activity and release [63-65], systemically administering their antagonists may affect serotonin transmission in multiple ways, not only reducing postsynaptic receptor activation but also increasing overall 5-HT signaling. Despite this complexity, behavioral changes resulting from blockade of these receptors partially mimicked those observed after 5-HT depletion, suggesting that the reduction in motivation was mainly due to decreased 5-HT signaling via 5-HT_1A_R and 5-HT_1B_R. Clear dissection of presynaptic regulatory mechanisms, especially in primate models, is challenging with currently available technology. Therefore, further investigation using advanced/innovative methods will provide deeper insights into the intricate interplay between 5-HT signaling and receptor subtypes." (in Discussion; lines 433–442)*

It is difficult to work out exactly how many datasets were collected for each part of the study. Please add to the main manuscript an overview table to show which manipulations / measures were collected in which monkeys.  

R: In response this, and similar comments from reviewer 1, we have made supplementary table (S2 Table) that summarizes the number of manipulations and behavioral tests conducted for each individual monkey, allowing for a clear understanding of the data collection process. In addition, we have updated the corresponding text and figure legends in the Results section to refer to this table. We believe that these changes address the reviewer's concern about this issue.

Drug doses were targeted to achieve 30-40% receptor occupancy. Please include the rational for these occupancy levels. You do mention that you aim to achieve the same level of occupancy per drug, but does this mean that these are also the required doses to observe an effect? Could perhaps the absence of effects in some of the drugs (esp. 5HT4) be the result of a too-low occupancy (i.e. underdosing)?

R: Our rationale for targeting 30-40% receptor occupancy was grounded in our depletion study showing a significant increase in error rate accompanied by at least a 30% decrease in 5-HT metabolites (Figs. 1 and 2). By aiming for a similar level of occupancy for each receptor subtype, we sought to replicate the behavioral changes associated with serotonin depletion and to investigate the involvement of specific receptors in decreased motivation. To clarify this rationale and its relevance to our observations, we have included these explanations in the Results section.

*"We first determined how much 5-HT is depleted by preventing its synthesis with pCPA. We repeatedly injected pCPA (150 mg/kg, s.c.) over the course of 2 days, which resulted in a decrease in 5-HT metabolites (5-hydroxyindole acetic acid, 5-HIAA) of at least 30% (N = 2, 33% and 64%) in the cerebrospinal fluid (CSF), while the concentration of DA remained unchanged (Fig 1A and 1B). The 30% value was later used when determining the dosages for specific 5-HT receptor-type antagonists." (in Results; lines 64–69)*

*"To mimic a reduction in 5-HT transmission comparable to the serotonin depletion observed in the CSF after pCPA treatment (approximately 30%), we measured tracer binding at baseline and after systemic administration in four monkeys (S1 Table; S1 Fig) and established the dose of antagonist required to achieve approximately 30%–40% blockade (receptor occupancy), except for 5-HT_2A_, which achieved over 50% (Table 1)." (in Results; lines 174–179)*

The reviewer also expressed concern that the 30% occupancy goal was too low and might have resulted in ineffective receptor blockade. However, blockade of 5-HT_4_R indeed significantly reduced error rates (Fig. 4C). In contrast, 5-HT_2A_R blockade produced no significant changes in error rates (Figure 4D). This might have been due to low dosages (despite a higher occupancy rate of 50%). In response to this concern about possible underdosing, we performed a preliminary test with a higher dose of the antagonist, which achieved over 80% occupancy (84%) (S2 Fig.). However, it had no discernible effect on the error rate in the reward size task. Therefore, we have updated the Results section to include these additional findings, which reinforce the notion that 5-HT_2A_R and 5-HT_4_R are unlikely to mediate the behavioral changes observed with 5-HT depletion.

*"In contrast, blockade of 5-HT_2A_R did not significantly affect either (Fig. 4C; Model #25, S5 Table). This was not because the blockade level was too low, as another high dose of the antagonist (achieving approximately 84% occupancy) did not change the error rates (S2 Fig). Blocking 5-HT_4_R consistently decreased error rates (Fig. 4D) and significantly increased a to approximately 150% (Fig. 4E; Model #30, S5 Table). Thus, it is unlikely that 5-HT_2A_R or 5-HT_4_R directly mediates the behavioral changes observed with 5-HT depletion." (in Results; lines 188–193)*

Please explain the tasks in more detail in the main manuscript, rather than referring to previous publications. both in the figure captions showing the task as well as the methods section. Please provide more detail as to how effortful this task is to the monkey, i.e. how strongly this task is tapping into effort processing. 

R: We have revised the legends of Figs 2 and 5, as well as the Methods section to provide a clear description of the task.

We also expanded the description of the task in the Results section to include the level of effort needed for the monkeys. In fact, the task itself is very easy for the monkeys to do, so they rarely fail when they actually try to perform it. For this reason, we use the error rate as a measure of motivation. We have added the following sentences to the Results section.

*"All monkeys had been trained to perform basic color discrimination trials on a cued multi-trial reward-schedule task [17] for more than 3 months. As in previous experiments using a single option task, the required action was very easy, and monkeys could not fail if they actually tried to release the bar at the proper time (the error rate is indeed much lower in the absence of information about costs and benefits) [2]. As in previous experiments in which costs and benefits were manipulated, errors (either releasing the bar too early or too late) were usually observed in small reward trials and/or close to the end of daily sessions [2, 18, 19]. Note that error trials were repeated with the same cue-reward condition, which prevented the monkeys from skipping unwanted trials. Therefore, bar “errors” were considered to have occurred when the monkeys were not sufficiently motivated to release the bar at a time that would lead to reward. The frequency of error trials is thus a reliable metric for quantifying the influence of motivation on behavior [15, 18, 19]." (in Results; lines 76­­–87).*

The serotonin literature cited is relatively old (most papers at ~ 20 years) and primarily focused on the delay discounting / waiting literature. I suggest to also link to the more recent theorizing on the role of serotonin in aversive inhibition and aversive 'pruning' by e.g. Quentin Huys, and relevant rodent optogenetics work by Cohen and colleagues on punishment and reward processing.

R: We thank the reviewer for the suggestion. In the revised manuscript, we have referred to the references suggested by the reviewer to discuss the relevance 5-HT’s role in aversion and punishment as follows:

*"Studies examining the activity of 5-HT neurons and their optogenetic manipulation also encourage us to consider motivation-independent factors beyond cost sensitivity. 5-HT neurons convey information not only about reward but also about aversive events [58, 59]. In addition, another example of complexity is the involvement of 5-HT in orchestrating behavioral inhibition [60]. Future investigation will be needed to examine the consequence of manipulating 5-HT transmission in nonhuman primates in the context of reward and punishment." (in Discussion; lines 405­­–410).*
