# Supplementary material for: Reduced serotonergic transmission alters sensitivity to cost and reward via 5-HT1A and 5-HT1B receptors in monkeys
Source: PLoS Biol. 2024 Jan 1;22(1):e3002445. doi: 10.1371/journal.pbio.3002445 (PMC10758260; doi:10.1371/journal.pbio.3002445)
Supplement: Attachment — Submitted filename: ResponseToReviewer1121.docx [file pbio.3002445.s018.docx]

Reviewer #3:

The authors comprehensively addressed most of my comments and clearly put in a lot of work. There are a few outstanding issues I would like to follow up on:

While I appreciate the Bayesian inference analysis (should be included in the paper) of the parameter robustness, I would still like to see parameter recovery. Note that my question here pertains to the model, and thus is independent of the sample size: even for a single set of parameters (i.e. 2 values of a and e) you can simulate e.g. 300 datasets and fit the model to those simulated datasets, and then report the average and spread of the parameter estimates fitted to the simulated data. You can repeat this procedure for each monkey / drug condition. This will give a good qualitative sense of how recoverable the parameters are.

R: We thank the reviewer for following up on this point. As per the reviewer's suggestion, we have included the results of the Bayesian inference analysis in S9 Fig. In response to the reviewer's additional request, we have performed a parameter recovery analysis, confirming that parameters *a* and *e* were successfully recovered for all conditions and monkeys, as shown in the figure below as a representative example.

**Figure. The parameter recovery for the best model of the data for 5-HT_1A_R blockade and control in monkey KN.** The red and green dots represent the parameter obtained from the model fitted to the average simulated data (4 sessions) generated from the mean and SD of the original data of 5-HT1AR blockade and control, respectively (N=1,000 times). The black points indicate the parameter obtained from the original data. Model #29 was used: err ~ x + (x | mk_cond).

Model comparison: if I understand correctly, in model 4 the random effects of a and e were coupled, while in model 3 they were independent? This means that if a went up by amount X, then e would go up by the same amount X? It would be good to make this even clearer in the text. Also in talbe S3, the notation of the model is exactly the same: E = 1/a(cond) R + e(cond), so it is unclear what the difference is between these models.

R: Thank you for pointing this out. The random effects of model #3 are assumed to be normally distributed on a and e, independently, while the random effect of model #4 is assumed to be a single two-dimensional normal distribution on a and e. We have revised Table S3, the legend, and the corresponding main text (line 103) to clarify these differences.

| Model | | BIC | | ΔBIC | |
| --- | --- | --- | --- | --- | --- |
| #1 | *E = 1/a(cond) R* | | 29.2 | | 13.3 |
| #2 | *E = 1/a(cond) R + e* | | 25.2 | | 9.3 |
| #3 | *E = 1/a(cond) R + e(cond)* | | 18.1 | | 2.2 |
| **#4** | ***E = 1/a(cond) R + e(cond),  　　　with (1/a(cond), e(cond)) ~ biNorm*** | | **15.9** | | **0** |
| #5 | *E = 1/a R + e(cond)* | | 22.2 | | 6.3 |

I am happy to see that the authors have updated their model comparison approach with the BIC. However, I would strongly suggest to report both AIC and BIC, as these offer a sort of 'upper' and 'lower' bound on how much to penalize for model complexity (in reality BIC is often too strict as parameters are rarely fully independent). The difference in model evidence for the models 3 and 4 is only 2.2, which is substantially lower than what is normally considered to be strong evidence in favour of one model over another. This is indeed confirmed by the fact that for the AIC, the preferred model is model 3. In all, this points to the fact that one just cannot draw a strong conclusion in favour of either model 3 or 4. Thus, I would like to see both he AIC and BIC reported in table S3, and the conclusion to be updated that there is just not strong evidence in favour of either model. I do agree with the approach of the authors that the most 'conservative' conclusion is to go with model 4 as there is at least no strong evidence for a double dissociation.

R: Again, we thank the reviewer for the suggesting the use of BIC and for the positive comments on our changes. The reviewer's additional comments seem to be based on a lack of clarity about the differences between models #3 and #4 in our manuscript, as pointed above. We would like to emphasize that both models #3 and #4 assume a significant effect of treatment on both a and c. We would also like to point out that both AIC and BIC consistently favor model #4, as shown below. Therefore, we believe that these results provide compelling evidence to support our conclusion that serotonin depletion resulted in two patterns of change in error rates. We believe that these clarifications address the reviewer's concerns.

| Model | | BIC | ΔBIC | AIC | ΔAIC |
| --- | --- | --- | --- | --- | --- |
| #1 | *E = 1/a(cond) R* | 29.2 | 13.3 | 27.8 | 14.8 |
| #2 | *E = 1/a(cond) R + e* | 25.2 | 9.3 | 23.2 | 10.2 |
| #3 | *E = 1/a(cond) R + e(cond)* | 18.1 | 2.2 | 15.7 | 2.7 |
| **#4** | ***E = 1/a(cond) R + e(cond),   with (1/a(cond), e(cond))~biNorm*** | **15.9** | **0** | **13.0** | **0** |
| #5 | *E = 1/a R + e(cond)* | 22.2 | 6.3 | 20.2 | 6.8 |

For figure 5, I don't see a reason to not simply present the data from all monkeys, to give the reader a complete overview of the (variability of) the effects. I would therefore like to see the data for all monkeys that were tested for each condition - these can just be colour (or saturation) -coded and included in the same graph.

R: We thank the reviewer for this suggestion. We agree that it is worthwhile to present data from all monkeys for Figure 5. Because showing all the data in one figure was too much, we have plotted the data from other subjects separately in Fig. S6.
